# Piezoresistive Properties of 3D-Printed Polylactic Acid (PLA) Nanocomposites

**DOI:** 10.3390/polym14152981

**Published:** 2022-07-22

**Authors:** Razieh Hashemi Sanatgar, Aurélie Cayla, Jinping Guan, Guoqiang Chen, Vincent Nierstrasz, Christine Campagne

**Affiliations:** 1 Textile Materials Technology, Department of Textile Technology, Faculty of Textiles, Engineering and Business, University of Borås, SE-501 90 Borås, Sweden; vincent.nierstrasz@hb.se; 2 ENSAIT, ULR 2461—GEMTEX—Génie et Matériaux Textiles, Université de Lille, F-59000 Lille, France; aurelie.cayla@ensait.fr (A.C.); christine.campagne@ensait.fr (C.C.); 3 College of Textile and Clothing Engineering, Soochow University, Suzhou 215006, China; guanjinping@suda.edu.cn (J.G.); chenguojiang@suda.edu.cn (G.C.)

**Keywords:** piezoresistive properties, 3D printing, fused deposition modelling (FDM), polylactic acid (PLA), multi-walled carbon nanotubes (MWNT), high-structured carbon black (KB)

## Abstract

An increasing interest is focused on the application of 3D printing for sensor manufacturing. Using 3D printing technology offers a new approach to the fabrication of sensors that are both geometrically and functionally complex. This work presents the analysis of the 3D-printed thermoplastic nanocomposites compress under the applied force. The response for the corresponding resistance changes versus applied load is obtained to evaluate the effectiveness of the printed layer as a pressure/force sensor. Multi-walled carbon nanotubes (MWNT) and high-structured carbon black (Ketjenblack) (KB) in the polylactic acid (PLA) matrix were extruded to develop 3D-printable filaments. The electrical and piezoresistive behaviors of the created 3D-printed layers were investigated. The percolation threshold of MWNT and KB 3D-printed layers are 1 wt.% and 4 wt.%, respectively. The PLA/1 wt.% MWNT 3D-printed layers with 1 mm thickness exhibit a negative pressure coefficient (NPC) characterized by a decrease of about one decade in resistance with increasing compressive loadings up to 18 N with a maximum strain up to about 16%. In the cyclic mode with a 1 N/min force rate, the PLA/1 wt.% MWNT 3D-printed layers showed good performance with the piezoresistive coefficient or gauge factor (G) of 7.6 obtained with the amplitude of the piezoresistive response (*A_r_*) of about -0.8. KB composites could not show stable piezoresistive responses in a cyclic mode. However, under high force rate compression, the PLA/4 wt.% KB 3D-printed layers led to responses of large sensitivity (*A_r_* = −0.90) and were exempt from noise with a high value of G = 47.6 in the first cycle, which is a highly efficient piezoresistive behavior.

## 1. Introduction

In recent years, 3D printing, also known as additive manufacturing (AM), has attracted significant attention from both industry and academia. In this technique, different methods such as material jetting [1], powder bed fusion [2], material extrusion [3], sheet lamination [4], directed energy deposition [5], photopolymerization [6,7], and binder jetting [8] are applied for the manufacturing of 3D items. These methods begin with a 3D model of the object, and then the special software digitizes and slices the object into the model layers. Afterward, the AM system prints 2D layers into a 3D build [9,10,11,12].

Three-dimensional printing is a novel method for the development of multifunctional components such as sensors with complex geometrics and combined characteristics such as optical, chemical, electrical, and thermal, etc. [9]. It is possible to embed a sensor into a 3D-printed component or print the entire sensor consistently [13]. In the recent past, significant research has been carried out on the fabrication of 3D-printed sensors such as force [14], motion [15], optic [16], hearing [17], etc. by various 3D printing techniques with distinctive transduction mechanisms, applications and printing materials. 

Fused deposition modeling (FDM) is one of the key AM methods, in which a thermoplastic filament is passed through a heated extrusion nozzle to be melted. Saari et al. [18] created a capacitive force sensor using the FDM method and ABS (Acrylonitrile butadiene styrene)-based materials consisting of a 3D-printed rigid frame with embedded wires in a spiral pattern imitating a flat plate capacitor and a thermoplastic elastomer dielectric spacer that compress under the applied force. An ear prosthesis fabricated by 3D printing of polyvinylidene fluoride (PVDF) [19] showed reliable responses under different conditions of pressure (0 to 16,350 Pa) and temperatures (2 to 90 °C) regarding the pyroelectric and piezoelectric properties. Krachunov [20] presented a novel method using 3D printing of ABS and polylactic acid (PLA) with silver coating for the design and manufacture of customized dry electrodes for Electroencephalography (EEG), which is a procedure that records brain activity in a non-invasive manner. The performance of the proposed electrodes is suitable for Brain–Computer Interface (BCI) applications, despite the presence of additional noise.

Applying electrically conductive polymer composites (CPCs) in FDM technology, some researchers have recently tried to develop sensors that are responsive to different stimuli such as chemicals including solvents, biological fluids, dopamine, serotonin, metals, vapors, mechanical flexing and liquid levels [21,22,23,24]. Kim et al. [25] 3D-printed thermoplastic polyurethane (TPU) and TPU/multiwalled carbon nanotubes (MWNT) (a structural part and a sensing part, respectively) to fabricate a 3D multiaxial force sensor that could detect the submillimeter scale deflection and its corresponding force on each axis.

TPU containing MWNT/graphene was used to develop flexible strain sensors [26,27]. The results demonstrated TPU nanocomposites as an excellent piezoresistive feedstock for 3D printing with the potential for wide-ranging applications in soft actuators, feedback from high-speed robotic applications and 3D-printed wearable devices.

Polylactic acid (PLA) has attracted researchers to apply this biodegradable thermoplastic as the matrix polymer in 3D-printed sensors. The total volatile organic compounds and ultrafine particles emitted while PLA printing is lesser in comparison to other polymers [28]. Three-dimensional printed PLA-carbon black could be effectively used as solvent [29] and capacitive sensors [30]. The tensile and impact strengths decreased after dipping them in solvents. The research showed that 3D-printed PLA containing nanographite/graphene is a promising economical electrochemical sensing platform; however, the performance of the 3D-printed devices is needed to be improved by increasing the percent of active material [31,32,33].

Printing in different geometries expands the utility of 3D-printed sensors in wearable forms and brings researchers closer to the desire of applying 3D printing for functional and smart textiles [34]. However, the existing high-sensitive pressure sensors in the medium- to high-pressure range could not be simply integrated into the garments without hindering the manual motion [35,36]. The sensors with a sensitivity in the medium pressure range (10–100 kPa) are required in gloves for monitoring hand stress during manual activity and object manipulation [37,38]. Foot pressure due to body weight as well as the applied force in using tools such as tennis rackets with repetitive motions are other examples of the medium pressure range [37]. Dios et al. investigated the piezoresistive performance of polymer-based nanocomposites in walking detection applications. Poly(vinylidene fluoride) (PVDF) in comparison with styrene-b-(ethylene-co-butylene)-b-styrene (SEBS) and thermoplastic polyurethane (TPU) is the most suitable polymer matrix in low deformation applications, whereas TPU and SEBS are suited for large deformation application due to their stretchability [39].

In fact, the piezoresistivity of 3D-printed PLA nanocomposites has not been investigated. Although the matrix is not flexible, the 3D-printed structure could bring functionality to the nanocomposites through possible complex geometries and the layer-by-layer structure which causes the inter-fillers and inter agglomerates gap to increase, leading the conductive nanocomposites to less dense and more sensitive to compression.

Therefore, in this research, the behavior of PLA 3D-printed nanocomposites under a load in the medium pressure range was investigated. Conductive filaments including multi-walled carbon nanotubes (MWNT) and high structured carbon blacks (Ketjenblack) (KB) in a PLA matrix were 3D printed and the electrical and compressive piezoresistive behavior of 3D-printed components were investigated. The piezoresistive behavior under compression was also studied in a cyclic mode in terms of filler type, filler content, and loading force rate.

## 2. Materials and Methods

### 2.1. Materials

As an electrically insulating thermoplastic matrix, a semi-crystalline polylactic acid (PLA) was purchased from NatureWorks, Minnetonka, MN, USA under the reference NatureWorks^®^-6202 D (Mn = 58,300 g/mol; D-Isomer = 1.3%). Prior to compounding and extrusion, PLA pellets were dried at 60 °C for 12 h in oven to remove water.

The carbon black (KB) was obtained from AKZO NOBEL, Amersfoort, The Netherlands under the reference Ketjenblack^®^ EC-600JD with the aggregate size of 10–50 nm, the apparent bulk density of 1–1.2 g/cm^3^ and BET surface area of 1400 m^2^/g. Multi-wall carbon nanotubes (MWNTs) were obtained from Nanocyl, Sambreville, Belgium under the reference Nanocyl^®^-7000 with a diameter of about 10 nm and lengths of 0.1–10 µm with a surface area of 250 m^2^/g.

### 2.2. Nanocomposites Preparation

In first step, a Thermo Haake co-rotating intermeshing twin-screw extruder was used to disperse fillers (MWNT or KB) into PLA with a weight percentage of 10 wt.%. The screw size of Haake is 400 mm in length and an average diameter of 16 mm (L/D = 25). The pressure is about 20 bar. The rotational speed of the screw was set at 100 rpm and the temperature of the five heating zones of the extruder was set at 160, 175, 175, 170 and 160 °C. Upon exiting the extruder with an average speed of 1 m/min, the masterbatch was pelletized. In the following step, the pelletized masterbatch was diluted with PLA pellets to obtain the weight percentage of 0.5–5 wt.% for MWNT and 1.5–7 wt.% for KB in PLA. Before dilution, both pelletized masterbatch and PLA pellets were dried at 60 °C for 12 h. For cooling down the manufactured filaments, a bath of closed circulation of water at room temperature was applied. The developed 3D printer filaments were used to print the nanocomposite layers.

### 2.3. 3D Printing

The 3D printer used (a two-head WANHAO Duplicator 4/4x) supplied by Creative Tools AB (Halmstad, Sweden) with a nozzle diameter of 0.4 mm and maximum printing size of 22.5 × 14.5 × 15 cm^3^. The 3D models were created in Rhinoceros software and exported as an STL (Standard Triangle Language that is the industry standard file type for 3D Printing), then transferred to Simplify3D software (Creative Tools AB) to be printed. Samples were 3D printed in different geometries including rectangular for electrical resistance measurement (1 × 12.75 × 60 mm^3^) and circular for compression (1 mm thickness and 40 mm diameter) at 240° ± 2 °C. The raster angle was 0° with linear infill pattern (100%). The raster size was 0.3 mm in height and 0.4 mm in width. The printing speed was 3000 mm/min and the first layer speed was 50%.

### 2.4. Electrical Resistance Measurement

The electrical resistance of 3D-printed layers was measured using a two-point measurement method by a digital multimeter connected with alligator clips to rectangular 3D-printed layers (1 × 12.75 × 60 mm^3^). Three measurements per CPC formulations (PLA/2, 3, 4, 5, 7 wt.% KB) and (PLA/0.5, 1, 1.5, 3, 5 wt.% MWNT) were carried out.

### 2.5. Piezoresistive Pressure Measurement

**Figure 1:**
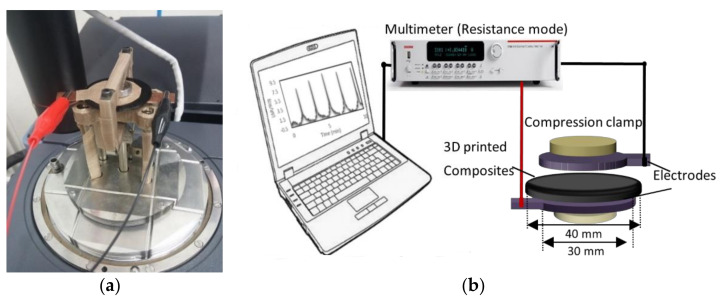
(**a**) Sample setup applied to investigate the piezoresistive properties of 3D-printed nanocomposite layers under compressive stress; (**b**) Schematic diagram of the positioning of the sample in clamps and electrodes.

The piezoresistive properties of the 3D-printed samples under compression were measured by compression clamp (15 mm diameter) consisting of DMA Q800 and a multimeter/system switch (Keithley 3706A) controlled by the instrument web interface. Figure 1 describes the experimental setup applied to investigate the piezoresistive properties of the samples. The 3D-printed layers with a thickness of 1 mm and a diameter of 40 mm were clamped between two copper plates of 30 mm diameter as electrodes to investigate the piezoresistive behavior. Copper plates were connected to a multimeter to measure the nanocomposite layers’ resistance. The samples and electrodes were clamped between a fixed part and the moving part providing the force (a Teflon tape was used for fixation). An initial preload of 2 N was applied to the sample in order to ensure full contact between the loading clamps and the sample surfaces. Then, compressive stress was used in the direction of resistance measurement to the sample. The geometry of the sample changes continuously due to the applied stress. Compressive loading was applied during the test at two different force rates (1 and 18 N/min) up to 18 N. DMA compression clamps yielded increasing pressure on the electrodes providing responses in the form of resistance.

The piezoresistive response (*A_r_*) or relative difference of resistance amplitude of sensors was calculated according to Equation (1) [40]:

(1) $$A_{r}=\frac{\Delta R}{R_{0}}=\frac{R-R_{0}}{R_{0}}$$

where *R* represents the resistance of the composite under applied pressure and $R_{0}$ is the static resistance.

## 3. Results and Discussion

### 3.1. Electrical Characterization

The conductivity (σ) of the 3D-printed layers was calculated according to Equation (2):

(2) σ=LR·A

where *L* and *A* are, respectively, the length (m) and the cross-sectional area (m^2^) of the 3D-printed layers. *R* is the electrical resistance (Ω) and σ is the electrical conductivity (Ω·m)^−1^ or Siemens per meter (S/m).

**Figure 2:**
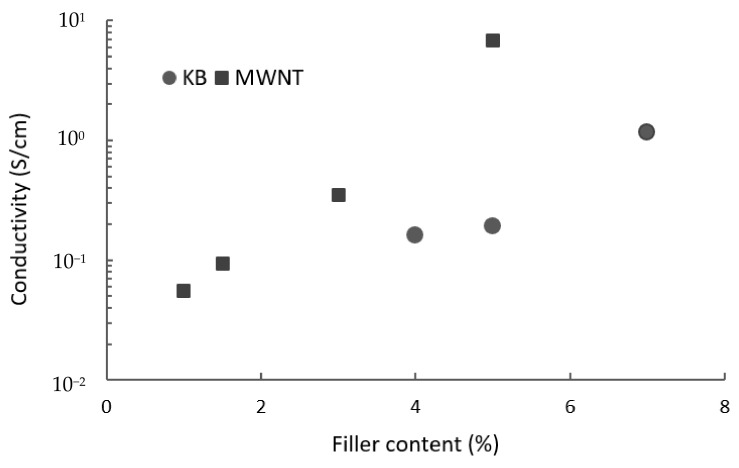
Electrical conductivity as a function of the filler content for 3D-printed layers of PLA nanocomposites containing MWNT and KB.

The sudden transition from insulator to conductor, which is the indication of the percolation threshold happened in PLA/4 wt.% KB and PLA/1 wt.% MWNT (Figure 2). The printed nanocomposites containing 2 and 3 wt.% KB as well as 0.5 wt.% MWNT were not conductive.

### 3.2. Compression Piezoresistive Properties

**Figure 3:**
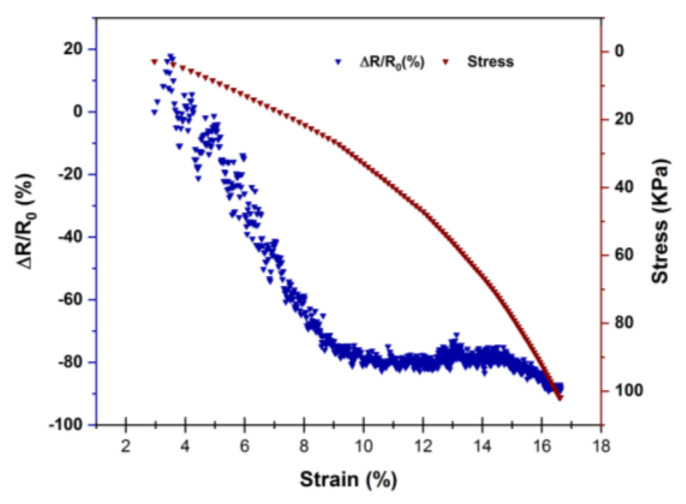
Piezoresistive responses of 1% MWNT nanocomposite 3D-printed layers under compressive loading.

The piezoresistive behavior of 1% MWNT 3D-printed nanocomposite layers was investigated when subjected to compression stress ranging from 0.5 to 18 N. Figure 3 represents the piezoresistive source signals evolution and the related stress–strain diagrams. It is evident that the 1 wt.% MWNT 3D-printed composite layer shows a negative pressure coefficient (NPC) characterized by a decrease of about one decade in resistance with the compressive loadings increase up to 18 N with the maximum strain up to about 16%. Piezoresistive pressure sensors undergo a change in resistance under applied pressure that is assumed to be caused by the different compressibility of filler and polymeric matrix under an applied force. Fillers either separate or approach the applied compression and cause a positive or negative relationship between pressure and resistance depending on filler geometry and the magnitude of the pressure [41]. The 3D-printed layers approach by the applied compression causing to more effective connections between conductive nanofillers by decreasing the average inter-fillers distance and hence lower relative resistance, which describes the NPC effect detected in the 3D-printed nanocomposites. The layer-by-layer structure of the 3D-printed nanocomposites causes the inter-fillers and inter agglomerates gap to increase, leading the conductive nanocomposites being less dense and more sensitive to compression.

### 3.3. Compression Piezoresistive Behavior in a Cyclic Mode

**Figure 4:**
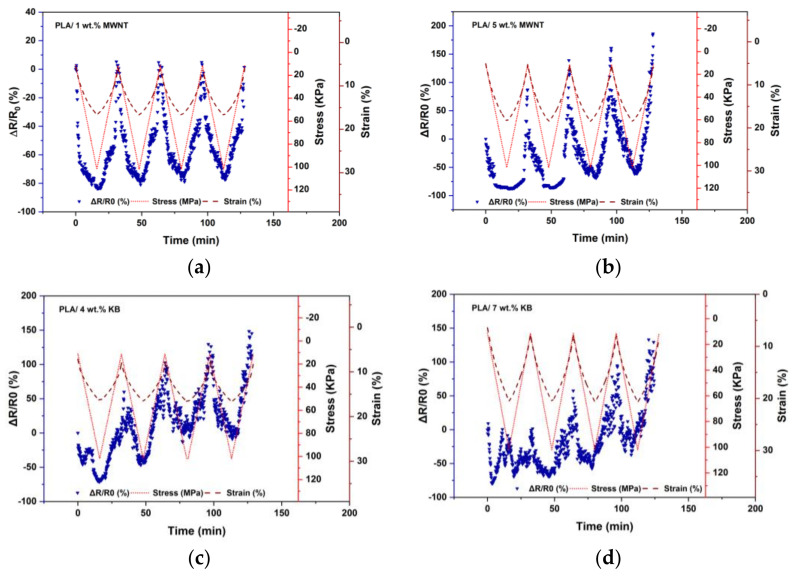
Comparison of 3D-printed nanocomposites piezoresistive responses: (**a**) PLA/1 wt.% MWNT, (**b**) PLA/5 wt.% MWNT, (**c**) PLA/4 wt.% KB and (**d**) PLA/7 wt.% KB.

A resistance change of the 3D-printed nanocomposite layers was detected under cyclic loadings increasing from 10 to 100 kPa. Four cycles were carried out to check the reproducibility of the sample with different filler contents (a sample in percolation threshold and a sample with higher contents of fillers) in low force speed of 1 N/min and a 15 mm diameter compression clamp. Figure 4 illustrates the sensors’ responses to applied stress and related strain.

**Figure 5:**
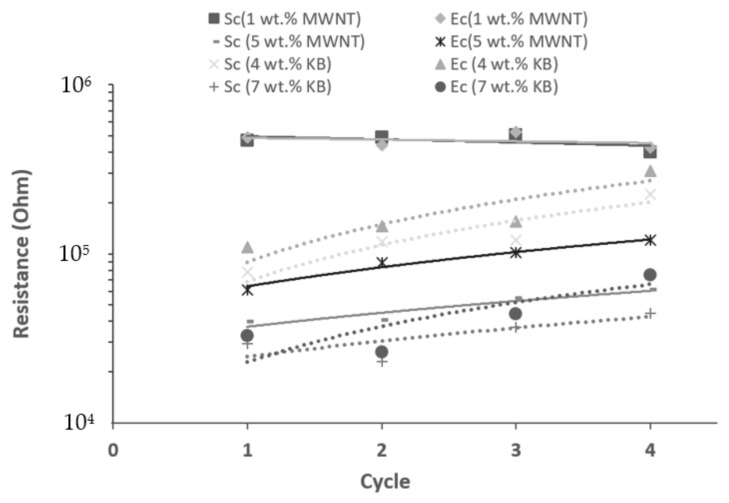
Resistance changes at the start and end of each cycle for different samples (Sc is the start of the cycle and Ec is the end of the cycle. The solid and dot linear trend lines represented MWNT and KB composites, respectively.

As shown in Figure 4a, PLA/1 wt.% MWNT piezoresistive responses are synchronic with strain and stress and the resistance variation follows the deformation which turns back to its original value after unloading. However, the sensor responses of samples including 5 wt.% MWNT, 4 and 7 wt.% KB are not synchronic with the applied stress and strain. Figure 5 represents the resistance changes at the start (Sc) and end (Ec) of each cycle for all samples.

It is clear that except for the PLA/1 wt.% MWNT, the other 3D-printed layers have significant hysteresis behavior, which is because of the residual strain of the 3D-printed layer composites after the compression. The same behavior was reported in studies about the compression test of porous structures including carbon nanotubes [42,43]. The melt flow index of composites with higher filler contents is low [44], therefore, it is required to 3D print at a lower speed or use a higher nozzle temperature [45], which causes structures with an eventually larger hysteresis behavior under compression cycles. Moreover, in higher nanofiller contents than the percolation threshold, the dominant mechanism of conduction is percolation [46], therefore, the destruction of effective conductive paths in successive loading/unloading cycles is the dominant mechanism, especially when the related strain is also high (low force speed). Therefore, by successive loading/unloading cycles in higher filler contents, an increase in minimum and maximum sensitivity is observed.

**Figure 6:**
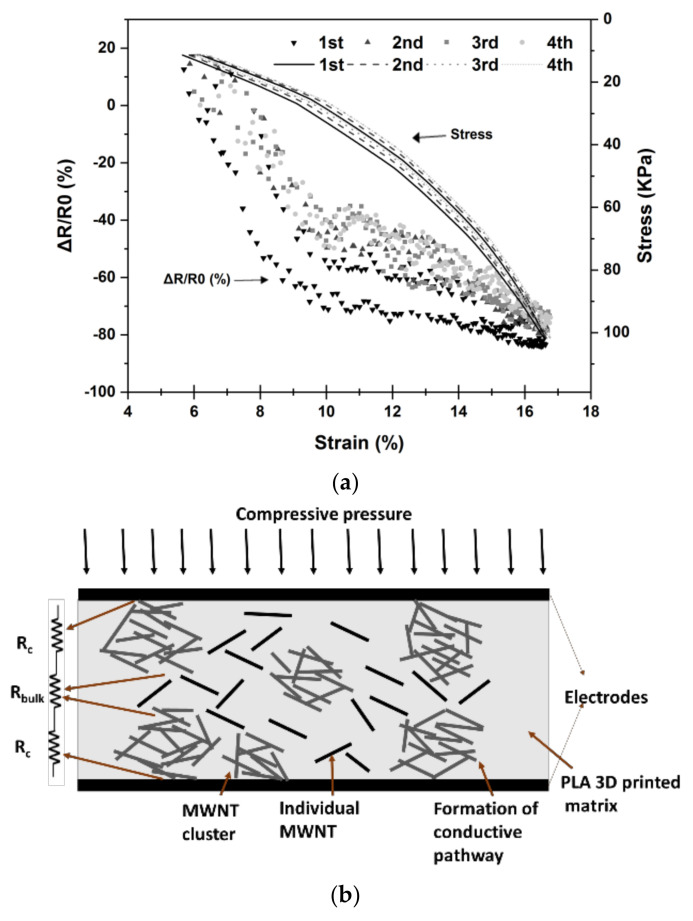
Piezoresistive behavior of 3D-printed PLA/1 wt.% MWNT nanocomposite: (**a**) Synchronism of *A_r_* with stress versus deformation. (**b**) Schematic diagram of the transduction mechanism of PLA/1 wt.% MWNT nanocomposite sandwiched between two metal electrodes towards compressive pressure. The electrical model of the FSR consists of a series of connections between the bulk (tunneling) resistance (*R_bulk_*) and the contact resistance (*R_c_*).

The hysteresis behavior of the PLA/1 wt.% MWNT layers is clearer in Figure 6, which shows that after a large hysteresis in the first cycle, the track of the three successive cycles of loading/unloading is nearly identical, with a minor deviation distinguished between the second and the fourth loops. This behavior is similar to the findings of Slobodian and Saha [47], where accordingly in the MWNT network, a ratcheting strain (mean value of the maximum and minimum strain in one cycle) takes place after the first compression cycle as a consequence of the primary deformation of the porous composition and blocked the reverse mobility of nanotubes in the middle of the dense networks. Through successive cycles of loading and unloading, the nanotubes’ reorder becomes stable and the MWNT network gets to a steady stress–strain hysteresis loop order. This suggests that when the carbon nanotube network is well deformed, it can be applied as a sensing component of compression stress. In Figure 6, it can also be observed that the signal is linear with a slope difference below and over 30 kPa. Figure 6b depicts a schematic representation of a 1 wt.% MWNT 3D-printed layer sandwiched between two copper electrodes. Dashed lines between the MWNT individual particles and clusters represent quantum tunneling bridges which accordingly allow charge carriers to tunnel from one cluster to another without any physical contact in composite systems.

When the sample is subjected to external stress (*σ*), the inter-particle distance is reduced. According to the proposed model by Paredes-Madrid et al. [46], the total resistance across the Force Sensing Resistor (FSR) can be decomposed from Equation (3):

(3) $$R_{FSR}=R_{bulk}+2R_{c}$$

where *R_bulk_* is the resistance of the CPC caused by the quantum tunneling phenomenon and *R_c_* is the contact resistance between the conductive particles and the metal electrodes. An FSR is created by the series connection between *R_bulk_* and 2*R_c_* as shown in Figure 6b. However, three phenomena occur when incremental stress is applied to an FSR [46]: (1) the contact resistance of the existing paths is decreased according to power laws; (2) new contact paths are constructed to a greater extent contributing to a decrease in the contact resistance; and (3) the average inter-particle distance is decreased, as a consequence decreasing the tunneling resistance, *R_bulk_*. It seems that for the 1 wt.% MWNT 3D-printed layer, the contact resistance is decreased by forming new contact paths and decreasing the contact resistance of the existing paths under 30 KPa and 10% strain. However, over 30 KPa, the resistance decreases because of the diminishing of inter-particle distance and consequently decreasing the tunneling resistance. The piezoresistive coefficient, which is also called the gauge factor, *G*, can be graphically figured out from the slope of the curve in Figure 6 and calculated with Equation (4) [40]

(4) $$G=\frac{A_{r}}{\varepsilon }$$

where $A_{r}=\frac{\Delta R}{R_{0}}$ (Equation (1)) is the piezoresistive response and ε$=\frac{\Delta L}{L_{0}}$ is the deformation of the sensor. For the 3D-printed PLA/1wt.% MWNT layers, the value of *G* = 7.6 was obtained with the amplitude of the piezoresistive response of about *A_r_* = −0.8 (−80%).

**Figure 7:**
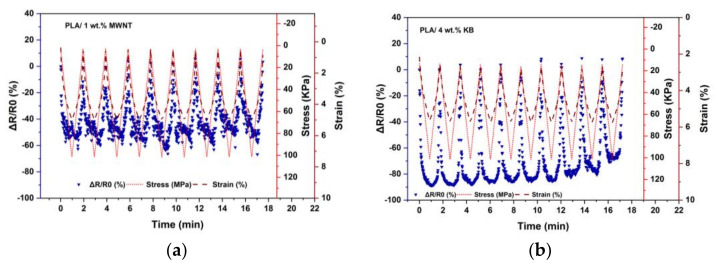
Comparison of 3D-printed nanocomposites piezoresistive responses in high force rate of 18 N/min (**a**) PLA/1 wt.% MWNT (**b**) PLA/4 wt.% KB.

To find out more about the sensitivity limitations of the developed FSR such as stress rate and related strain, the piezoresistive response of the 3D-printed composite layers under cyclic compressive stress with a high speed of 18 N/min was observed. Figure 7 shows the piezoresistive responses of the PLA/1 wt.% MWNT and PLA/4 wt.% KB samples, exposed to ten cycles of compressive stress from 10 up to 100 kPa.

**Figure 8:**
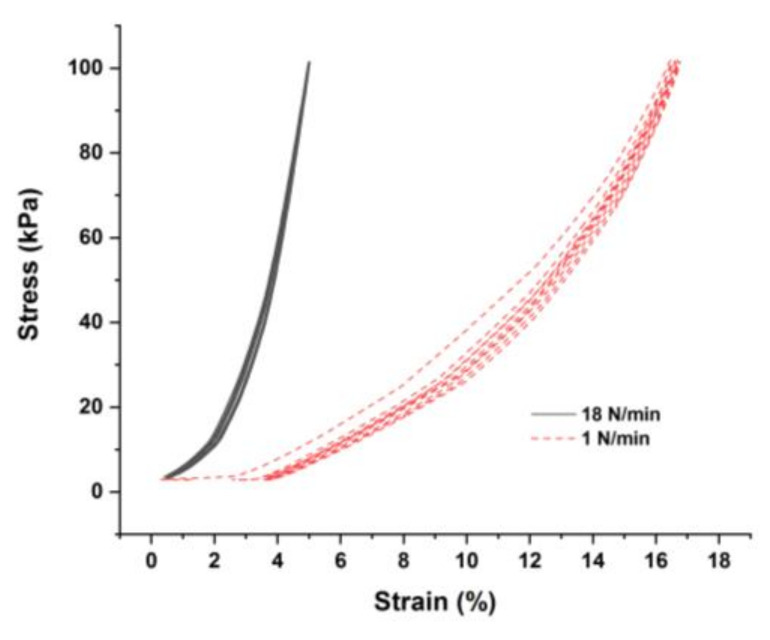
Stress–strain diagrams of 3D-printed PLA/1 wt.% MWNT with different force rates in a cyclic mode.

Figure 7a shows that the piezoresistive response of the PLA/1 wt.% MWNT with an applied force rate of 18 N/min has a smaller amplitude (*A_r_* = −0.60) and more noisy signals, but a higher value of *G* = 9.3 in comparison with low force rate of 1 N/min in Figure 4a. Figure 8 shows that applying a high force rate causes a smaller strain as there is insufficient time for the material to respond to stress with large-scale viscoelastic deformation or yielding [48].

In Figure 7b, the compression with a high force rate leads to responses of large sensitivity (*A_r_* = −0.90) and exemption of noise for the PLA/4 wt.% KB 3D-printed layers. However, after almost seven cycles, the maximum sensitivity is not stable and starts to decrease. The high value of *G* = 47.6 in the first cycle shows the high piezoresistive properties of these layers if the cyclic functionality is not needed. The gauge factor decreases to *G* = 28 in the 10th cycle of stress. Therefore, the PLA/KB 3D-printed layers do not show stable piezoresistive behavior in a cyclic mode at low and high force rates.

## 4. Conclusions

In this paper, fused deposition modeling 3D printing is used to develop CPC layers and investigate their electrical and piezoresistive behaviors. To this aim, multi-walled carbon nanotubes (MWNT) and high-structured carbon black (Ketjenblack) (KB) were incorporated into polylactic acid and 3D-printable filaments using the melt-mixing process were developed. The 3D-printed layers were created using fused deposition modeling. The percolation threshold of the MWNT and KB 3D-printed layers are 1 wt.% and 4 wt.%, respectively, by the two-point resistance measurement method. It was shown that it was possible to 3D print piezoresistive PLA nanocomposite layers from the MWNT and KB fillers and PLA matrix. The PLA/1 wt.% MWNT 3D-printed layers with a 1 mm thickness exhibit a negative pressure coefficient (NPC) characterized by a decrease of about one decade in resistance with increasing compressive loadings up to 18 N with a maximum strain up to about 16%. In the cyclic mode with a 1 N/min force rate, the PLA/1 wt.% MWNT 3D-printed layers showed good performance with a value of *G* = 7.6 obtained with the amplitude of the piezoresistive response of about *A_r_* = −0.8 (−80%). The response was linear in the range of pressure 10–100 kPa, with low noise and hysteresis that comes from the layer-by-layer architecture of the component and the tunneling effect of MWNT nanofillers in lower contents than the percolation threshold. At a high force rate of 18 N/min, the piezoresistive response of the PLA/1 wt.% MWNT has a smaller amplitude (*A_r_* = −0.60) and more noisy signals but a value of *G* = 9.3. The KB composites could not show stable piezoresistive responses in a cyclic mode. However, the PLA/4 wt.% KB 3D-printed layers under high force rate compression lead to responses of large sensitivity (*A_r_* = −0.90) and are exempt from noise with a high value of *G* = 47.6 in the first cycle. The results show that PLA/MWNT and PLA/KB can be considered good piezoresistive nanocomposites to be 3D printed where complex designs with functionality are needed for possible use in wearable electronics, soft robotics, and prosthetics, etc.

## Data Availability

Not applicable.
